# The influence of suturing and or gluing of perforated Schneiderian membrane during sinuslift procedure on the outcome: a retrospective study

**DOI:** 10.1186/s40729-024-00568-5

**Published:** 2024-11-04

**Authors:** Fouad Khoury, Christoph Schmidt, Jochen Jackowski

**Affiliations:** 1 Private Clinic Schloss Schellenstein, Am Schellenstein1, 59939 Olsberg, Germany; 2 Department of Oral and Maxillofacial Surgery, University of Münster, Münster, Germany; 3 Private Practice, Brilon, Germany; 4 Department of Oral Sugery and Dental Emergency Care, University of Witten, Herdecke, Germany

**Keywords:** Maxillary sinus, Sinus lift, Sinus grafting, Schneiderian membrane perforation, Fibrin glue, Membrane suturing

## Abstract

The sinus lift procedure has become the most common method for maxillary bone augmentation. The most frequently observed intraoperative complication is the perforation of the Schneiderian membrane. Various treatment options have been proposed for managing these perforations, including the use of resorbable membranes, centrifugated blood products as PRF, or PRGF, suturing, and fibrin glue application. While long-term studies exist for the use of resorbable membranes to close perforations, there is limited data on the long-term outcomes of suturing or gluing the perforated sinus membrane. The aim of this retrospective study is to evaluate the long-term outcomes of suturing and/or applying fibrin glue to repair perforated sinus mucosa during sinus floor elevation procedures. Between 2005 and 2009, a total of 692 patients underwent 923 sinus lift surgeries, and Schneiderian membrane perforation occurred in 202 sinus floor elevations (21.98%) across 168 patients. The main documented causes of perforations, which ranged from 2 to 10 mm in diameter, were the presence of septa, followed by thin and adherent membranes. Of the perforations, 100 (49.5%) were treated with microsurgical suturing combined with fibrin glue, 78 (38.6%) were treated with fibrin glue alone, and 24 (11.9%) were treated exclusively with suturing. Sinus grafting was performed using autogenous bone in combination with a biomaterial, following the layering technique. All surgeries resulted in primary healing without complications, enabling all patients to undergo restoration as planned. The long term clinical and radiological evaluations of 44 randomly selected patients who followed the recall program up to 10 years post operative confirmed the effectiveness of this treatment approach.

## Introduction:

Alveolar bone resorption after tooth loss can occur for various reasons. In the posterior maxilla, bone resorption may affect not only the crestal part of the jaw but also the basal bone borders near the maxillary sinus. This is largely due to respiratory overpressure, which leads to a pneumatization process, causing the maxillary sinus to extend toward the alveolar bone [1, 2]. These processes often result in the posterior maxillary alveolar bone being so hollowed out that only a paper-thin bone lamella separates the maxillary sinus from the oral cavity [3]. This physiological phenomenon of sinus pneumatization is influenced by stress, strain, and, notably, the presence or absence of teeth. The loss of maxillary molars reduces the resistance of the posterior maxilla's soft bone against respiratory overpressure, increasing the volume of the maxillary sinus. However, the degree of sinus expansion varies significantly between individuals, likely due to increased osteoclast activity beneath the Schneiderian membrane [3, 4].

As a result, bone augmentation is often required when planning implant placement in this region to create a suitable implant site [5]. Sinus floor elevation via the external lateral window approach is one of the commonly used bone grafting techniques for this purpose. It enables implant insertion either as part of a one- or two-stage procedure. This technique, aimed at increasing vertical bone height beneath the Schneiderian membrane in a cranial direction, has been routinely employed for decades. Due to its high success rate, this method is considered a highly predictable and effective procedure [5–8].

A key factor in its success—and in minimizing complications—is careful elevation of the sinus membrane without perforating it, thereby creating a space between the basal sinus bone and the membrane for the bone graft material. This elevation is carried out until it reaches the planned implant length. However, lifting the Schneiderian membrane from the sinus floor is not always straightforward and can be particularly challenging in cases of thin, adherent mucosa or the presence of sinus septa. In such situations, the sinus membrane may perforate, creating a connection between the maxillary sinus, the bony sinus floor, and the oral cavity. The reported incidence of Schneiderian membrane perforations during sinus floor elevation procedures ranges from 4.7% to 56.16% [9–16], with an average rate of 22.5%. If left untreated, such perforations can lead to dislocation of the bone graft material into the sinus cavity, resulting in various types of sinusitis. Additionally, contamination and infection of the grafted area may occur, leading to inflammatory processes such as fistulae, abscess formation, and eventual loss of the bone graft and implants [17–21]. Various techniques and materials have been proposed for closing Schneiderian membrane perforations, including collagen membranes, collagen sponges, Vicryl nets, and the Loma Linda pouch [22–28]. Suturing, fibrin adhesive, or a combination of both have also been employed [29–32]. Additionally, Platelet-Rich Plasma (PRP) and Platelet-Rich Fibrin (PRF) have been used to cover membrane perforations [33–36], as have various pedicled flaps. [37–39]

The aim of this retrospective study is to evaluate the short term as well as the long-term outcomes of closing perforated sinus mucosa with resorbable sutures and or fibrin glue. This study is focusing on somewhat this kind of treatment is effective against dislocation of augmentation material into the sinus and so preventing a sinusitis with the looseness of the grafted bone and the implants.

## Materials and methods

Patients treated between 2005 and 2009 for sinus floor elevation with documented intraoperative perforation of the Schneiderian membrane at an academic privat hospital were included in this study. All patients were over 18 years old and provided written informed consent for the surgery, as well as permission to use their collected data—including preoperative, intraoperative, and postoperative documentation—for scientific publications. The study adhered to the principles outlined in the Declaration of Helsinki and was approved by the university's ethics committee (No. 125/2018). This study was conducted following the STROBE (Strengthening the Reporting of Observational Studies in Epidemiology) guidelines (http://www.strobe-statement.org [39].

Preoperative and postoperative clinical and radiographic data, surgical procedures, and postoperative follow-up were collected for all patients. Additionally, 44 patients from this group, who were undergoing other treatments at the clinic, were clinically and radiologically examined. Clinical inspection, palpation, panoramic radiographs (Sidexis, Dentsply Sirona, Germany), and digital volume tomography (Galileos, Dentsply Sirona) were used for diagnosis.

### Surgical procedure

All procedures were performed by a single experimented and academic oral surgeon following a standardized protocol. When maxillary bone height was ≥ 5 mm, a one-stage procedure with simultaneous implant insertion was performed. In cases where bone height was < 5 mm or where there were significant horizontal or vertical crestal bone defects, a two-stage procedure was utilized. Patients received 1.2 g of amoxicillin or, in case of allergy, 600 mg of clindamycin intravenously 30 min before surgery. Most surgeries were performed under local anesthesia with conscious sedation (Midazolam), although general anesthesia was used for multiple bone augmentation procedures.

After making a crestal incision and reflecting a mucoperiosteal flap on the buccal side of the maxilla, bone chips were harvested from the sinus wall using a bone scraper (Safescraper, Meta Reggio Emilia, Italy). As the sinus wall thinned, and with partial exposure of the Schneiderian membrane, a small round diamond bur was used to create an 8 × 6 mm window. The "trap door" of bone was removed and used as autologous graft material. The sinus membrane was carefully elevated from the sinus floor, and partial septa were fractured and removed [40].

**Fig. 1:**
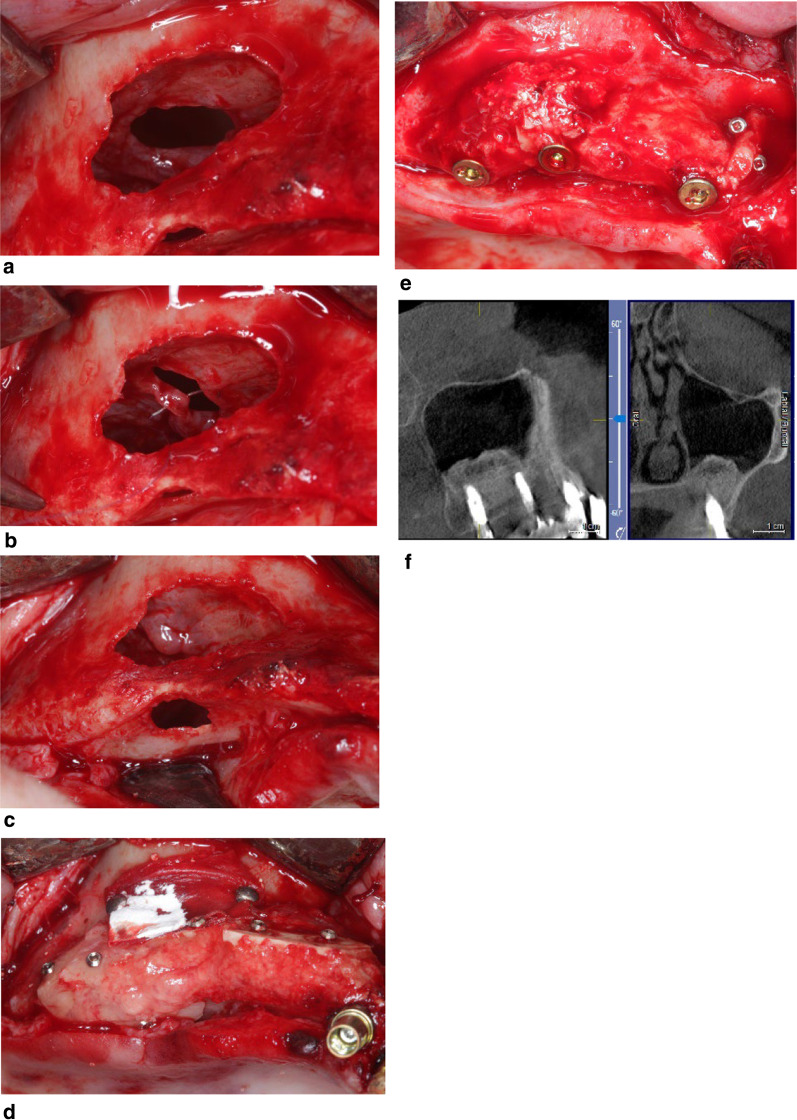
**a** A wide perforation of the Schneiderian membrane. **b** Suturing the 2 borders of the perforation with 70 resorbable sutures. **c **Clinical appearance after the hermetic closure of the perforated sinus membrane. A perforation of the bony basal border of the maxillary sinus is also present due to previous surgeries. **d** After grafting of the sinus floor, a3D bone augmentation of the alveolar crest was performed with autogenous bone grafts following the split bone block technique. **e** Insertion of 2 Implants in the grafted area after the 3 months healing period. **f** Control CBCT 10 years post operative confirming a stable grafted area with a free ostium and without any sinus pathology

**Fig. 2:**
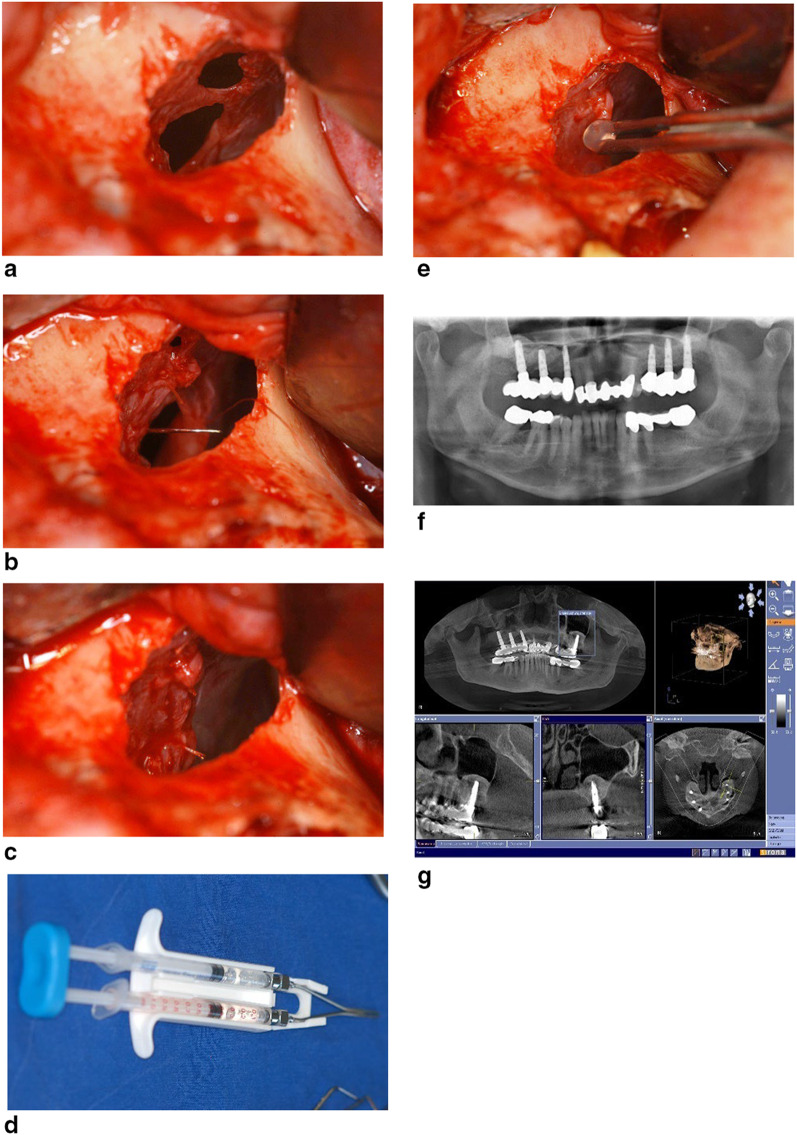
**a** Multiple perforation of the schneidarian membrane. **b** Suturing of the perforations with 70 resorbable sutures. **c** Hermetic closure of the perforations. **d** Fibrin glue syringe (Beriplast): 2 components joint together to form the adhesive. **e** The thin membrane is reinforced with the fibrin adhesive especially over the sutured membrane. **f** Control radiograph 10 years after the oral rehabilitation. **g** CBCT confirming the stability of the sinus graft around the osseointegrated implants with the free ostium and without any sinus pathology

In cases of Schneiderian membrane perforation, the elevation of the sinus mucosa continued mesially, palatally, and distally toward the tuber maxillae to mobilize as much sinus tissue as possible. This procedure aimed to bring the wound edges of the perforation closer together. The wound edges were then either sutured with resorbable microsurgical suture material made of polyglycolic acid (PGA Resorba 7–0, Resorba Medical GmbH, Germany) and/or glued with fibrin adhesive (Beriplast, CSL Behring GmbH, Germany). The choice between suturing and gluing depended on the anatomical situation, perforation location, and thickness of the sinus mucosa. Anterior perforations were typically sutured (Fig. 1a–f), while posterior perforations were more frequently glued due to limited access. Thin membranes were often both sutured and glued (Fig. 2a–g).

Once the Schneiderian membrane was sealed, it was further elevated to the desired height (approximately 15 mm). If residual bone height was ≥ 5 mm, simultaneous implantation was performed. The subantral space was filled with particulate autologous bone and biomaterial (Algipore, Dentsply Sirona) using a layering technique [41].

The sinus window was closed with the biomaterial and typically covered with a non-resorbable titanium membrane (Frios BoneShield, Dentsply Sirona Implants). Tension-free adaptation of the flap was achieved by releasing the periosteum. The mucoperiosteal flap was repositioned and sutured using resorbable sutures (Glycolon 6–0, Resorba). Antibiotics were continued for 7 days postoperatively (amoxicillin 2 × 1.2 g/day or clindamycin 2 × 600 mg/day). Post-augmentation care also included pain medication (Ibuprofen AL 400, Aliud Pharma), topical nasal decongestants (Otriven 0.1% nose drops, Novartis Consumer Health), regular mouth rinses (chlorhexidine 0.2%), and suture removal after 10 days.

Three to four months post-surgery, implants were either exposed or inserted if implantation had not yet occurred due to insufficient bone. In these cases, implants were exposed after an additional 3 months. Prosthetic restoration began 4 weeks later.

### Outcome measures

This study tested the result of suturing and or gluing of the perforated Schneiderian membrane during sinus lift procedures.

### Outcome measures were:

- Good healing of the surgical site, as determined clinically by primary healing of the soft tissue over the grafted area without tissue necrosis, suppuration, or bone exposure. The soft tissue must be normal in color without inflammation 2 weeks after surgery, the date of suture removal, and at re-entry.
- Good healing of the grafted sinus was clinically determined 3 months after surgery based on the normal color of the soft tissue without any pathology such as fistula, abscess or exposed bone as well as the absence of any sinusitis symptoms.
- The stability of the grafted area allowing the insertion of additional implants in case of 2 stage procedures.
- Implant failures such as implant mobility, removal of stable implants due to infection, or progressive marginal bone loss have been documented.
- In addition, the long-term radiological stability of the grafted sinus around the osseointegrated implants without any sinus pathology controlled on CBCT for the 44 Patients.

The result evaluation was not carried out by the operator and was therefore independent.

The Microsoft Exel program (Microsoft Office, Microsoft Corporation, Redmond, USA) was used to compile the statistics. This was sufficient for the calculation of mean values and standard deviations.

## Results

During the study period, 923 external sinus floor elevations were performed on one or both sides of 692 patients. The gender distribution was 62.86% female and 37.14% male. Among these patients, 162 (17.55%) identified as smokers.

A total of 202 Schneiderian membrane perforations (21.88%) occurred in 168 patients (59.5% female, 40.5% male), with perforation diameters ranging from 2 to 15 mm. The patients' ages ranged from 20 to 76 years, with an average of 57.71 ± 11,1 years. Smokers represented 34 of these patients (20.2%), with an average consumption of 13.6 cigarettes per day. In six cases, the Schneiderian membrane was ruptured multiple times. The causes of sinus membrane perforations were documented in only a few cases: in 55 cases (27.28%), perforations were caused by underwood septa, while in two cases (1%), a very thin membrane was the cause. For the remaining 145 cases, no specific cause was recorded. Preoperatively, 66 (32.7%) surgical sites had a residual bone height of less than 2 mm, 112 sites (55.5%) had a residual bone height of 2–5 mm, and 24 sites (11.9%) had a vertical residual bone height greater than 5 mm.

**Fig. 3:**
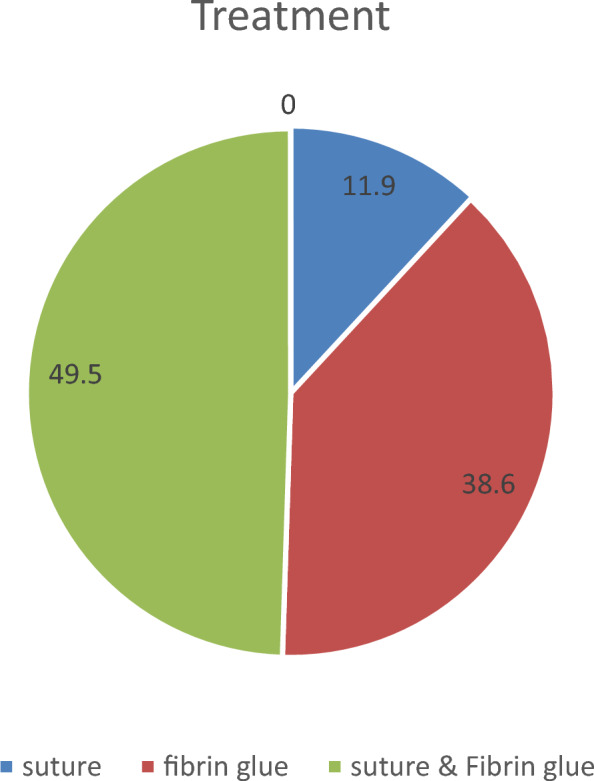
Prevalence of the different treatment options

Suturing alone with 7/0 resorbable material was performed in 24 cases (11.9%), fibrin adhesive alone was used in 78 cases (38.6%), and in the remaining 100 cases (49.5%), a combination of suturing and fibrin adhesive was applied (Fig. 3).

Simultaneous sinus floor elevation and implant placement of all planned implants were possible in 70 of the 168 patients (41.7%). In another 54 patients (32.1%), only a part of the implants could be primary placed, with the remaining implants inserted after graft healing during a second procedure, 3–4 months later. In the remaining 44 patients (26.2%), only sinus grafting was performed initially, with implant insertion scheduled for 3–4 months later (two-stage procedure).

Postoperative soft tissue complications, specifically small dehiscences, occurred at 5 surgical sites (2.5%). These complications had no effect on the outcome of the grafted sinuses or the simultaneously inserted implants. The dehiscences healed completely within 2 weeks following treatment with H₂O₂ and saline rinses. All grafted sinuses healed as expected, allowing for either implant placement or exposure of previously inserted implants 3–4 months postoperatively. A total of 5 of the 574 implants inserted (0.9%) were removed during implant exposure due to non-osseointegration, though there was no infection of the grafted area. In all cases, new implants were successfully inserted during the same session. The newly placed implants healed without any complications and were restored as planned. No further implant losses were recorded during the 5 to 10-year follow-up period.

Among the 168 patients with treated sinus membrane perforations, 44 remained under care at the clinic for other bone augmentation procedures at the time of this investigation and underwent clinical and radiological evaluation. The follow-up period for these 44 patients ranged from 5 to 10 years, with an average of 6.4 ± 2,1 years. CBCT scans were available for all 44 patients, encompassing a total of 57 treated sinus perforations. CBCT analysis showed no signs of sinus pathology, with a clear osteum in 50 sinuses (87.7%). The remaining 7 cases exhibited minor thickening of the sinus mucosa, but no clinical symptoms were present.

The vertical bone height, measured by CBCT on the day of the investigation, ranged from 12.8 mm to 24.7 mm, with an average height of 17.5 ± 2.48 mm.

## Discussion

The predominance of female patients (62.86%) in the investigated population is consistent with other studies [5, 15, 18]. This may be attributed to a greater desire for aesthetics and comfort, as well as a higher health awareness among women.

Sinus floor elevation is widely regarded as a safe procedure for creating sufficient bone volume in the posterior maxilla, enabling implant placement [4]. The main complication during surgery, besides significant bleeding, is perforation of the Schneiderian membrane [11, 13, 15]. If left untreated, this can lead to displacement of augmentation materials into the sinus, resulting in infection and procedural failure, as well as contamination and infection of the graft by sinus flora [39]. The reported frequency of Schneiderian membrane perforation during external sinus floor elevation ranges from 7 to 56%. This variation between studies is likely due to differing levels of surgical experience or failure to detect membrane perforation, often caused by not using a microscope or well-lit loupe during surgery. In the present study, the rate was 21.9%, which is comparable to most other authors' findings [10–13].

The most common predisposing factors for membrane perforation include a thin membrane and the presence of underwood septa [39, 41]. In this study, septa were documented as a predisposing factor in 27.3% of the perforations. Unfortunately, no other predisposing factors were recorded for the remaining surgeries. Thin and adherent Schneiderian membrane can have easily perforation during the elevation procedure which can compromise the outcome in case it was not closed hermetically [26, 27].

The sinus membrane has a high regenerative potential due to its rich vascularization, allowing it to repair in a short time. Various techniques and materials have been proposed for treating sinus membrane perforations. In addition to suturing with or without fibrin adhesive, and different forms of platelets, collagen membranes are the most commonly used technique [25–28]. This is because collagen membranes are easy to use, despite the lack of long-term results. However, suturing remains the gold standard for treating injuries of all kinds. Through suturing, it is possible to approximate the wound edges, allowing tension-free closure and secure healing. Fibrin adhesive is also a standard technique in general surgery where suturing is not feasible, such as in liver surgery, and has been successfully used for decades [42]. Fibrin glue is also commonly used in oral surgery for preventing and treating severe bleeding, especially in cases of hemorrhagic diathesis [39]

In this study, all sinus perforations were closed with resorbable sutures, fibrin adhesive, or a combination of both, depending on the situation and location. Perforations in the anterior part of the sinus were typically sutured due to easier access, while those in the posterior part, which were harder to reach, were usually treated with fibrin adhesive. For thin membranes, a combination of suturing and gluing was employed. This study demonstrated that all three techniques are highly predictable and reliable, with a very low complication rate compared to other authors [23, 25]. In the matter of fact, suturing of the Schneiderian membrane is not easy and needs good experimented surgeon to be able to close the thin membrane correctly. That was the case in the presented study where all the surgeries were performed by an experimented oral surgeon. On the other side, the use of fibrin adhesive is not difficult and can be used easily as alternative since the clinical and experimental studies confirmed the efficacity of such a treatment [29, 31, 32].

The use of collagen membranes to close Schneiderian membrane perforations, although widespread, is not physiologically ideal. Perforation of the sinus membrane creates a communication between oral and sinus flora. A collagen membrane covering both the perforation and the bone graft remains exposed to sinus flora after the grafting procedure, potentially leading to contamination and infection of both the membrane and the graft. Hyun-Chang Lim et al. found in a rabbit model that Schneiderian membrane perforation repaired using a collagen membrane delayed new bone formation in the augmented sinus [43]. Proussaefs et al. reported more than 30% complication rates in cases where perforated membranes were covered with collagen membranes [24, 25]. Choi et al. compared the use of autologous fibrin glue with collagen membranes for closing sinus membrane perforations during sinus lifts. In their experimental study, wounds repaired with autologous fibrin glue showed newly formed continuous epithelium across the perforation site, while wounds treated with collagen membranes exhibited extensive fibrosis, inflammatory infiltration, and absent epithelium [32].

The use of PRF or other growth factor membranes derived from autologous blood can also be considered a good alternative for closing sinus membrane perforations. Like fibrin glue, these membranes are relatively stable and elastic. Many studies have confirmed their positive role in improving soft tissue healing [33–36]. However, these membranes lack the adhesive effect needed to approximate wound edges and can only be placed over the perforation. This limits their use to small perforations, with the risk of slipping over or into the perforation during the grafting procedure.

The results of this study confirm that all three techniques used to treat sinus membrane perforation during sinus lift procedures—suturing, fibrin adhesive, and a combination of both—are equivalent and successful. The complication rate was very low and comparable to cases without membrane perforation: of the 574 implants placed in conjunction with a perforation, only five implant losses occurred (0.9%). No cases with fistula, Abscess or signs of acute or chronic sinus infection were observed. These results were also confirmed by clinical and radiographic (CBCT) examinations of 44 patients (Fig. 1f + 2 g). In five patients, a slight thickening of the maxillary sinus mucosa up to 6 mm was observed radiologically, though without significant clinical symptoms. Two of these patients reported frequent nasal congestion, a condition that had existed for many years before the surgical procedure. In one case, the perforation was sutured, while in the other it was sutured and glued. The remaining three patients reported no subjective limitations.

The results of this retrospective study are limited through the retrospective study design including incomplete data on the causes of sinus membrane perforation and potential biases due to the surgeon's technical proficiency with micro sutures. Less experienced clinicians could have more difficulties to suture correctly sinus membrane perforations. Other limitations are also due for the long-term results based on the clinical and radiographic results of only 44 patients.

## Conclusions

This study demonstrates that sinus floor elevation, even with intraoperative perforation of the Schneiderian membrane, is a very safe procedure when the perforation is properly and biologically sealed. The closure of a membrane perforation with resorbable sutures, fibrin adhesive, or a combination of suturing and gluing is highly recommended without reservation.

## Data Availability

No datasets were generated or analysed during the current study.
